# Contribution of rare and common species to subterranean species richness patterns

**DOI:** 10.1002/ece3.5604

**Published:** 2019-09-30

**Authors:** Petra Bregović, Cene Fišer, Maja Zagmajster

**Affiliations:** ^1^ SubBioLab Department of Biology Biotechnical Faculty University of Ljubljana Ljubljana Slovenia; ^2^ Croatian Biospeleological Society Zagreb Croatia

**Keywords:** amphipods, beetles, caves, conservation planning, Dinarides, endemism, range size

## Abstract

**Aim:**

Common species contribute more to species richness patterns (SRPs) than rare species in most studies. Our aim was to test this hypothesis using a novel model system, species living exclusively in subterranean habitats. They consist of mainly rare species (small ranges), only a few of them being common (large ranges), and challenge whether rare species are less important for the development of SRPs in this environment. We separately analyzed aquatic and terrestrial species.

**Location:**

Western Balkans in southeastern Europe.

**Methods:**

We assembled two datasets comprising 431 beetle and 145 amphipod species, representing the model groups of subterranean terrestrial and aquatic diversity, respectively. We assessed the importance of rare and common species using the stepwise reconstruction of SRPs and subsequent correlation analyses, corrected also for the cumulative information content of the subsets based on species prevalence. We applied generalized linear regression models to evaluate the importance of rare and common species in forming SRPs. Additionally, we analyzed the contribution of rare and common species in species‐rich cells.

**Results:**

Patterns of subterranean aquatic and terrestrial species richness overlapped only weakly, with aquatic species having larger ranges than terrestrial ones. Our analyses supported higher importance of common species for forming overall SRPs in both beetles and amphipods. However, in stepwise analysis corrected for information content, results were ambiguous. Common species presented a higher proportion of species than rare species in species‐rich cells.

**Main Conclusion:**

We have shown that even in habitats with the domination of rare species, it is still common species that drive SRPs. This may be due to an even spatial distribution of rare species or spatial mismatch in hotspots of rare and common species. SRPs of aquatic and terrestrial subterranean organisms overlap very little, so the conservation approaches need to be habitat specific.

## Graphical abstract

We studied whether species richness patterns develop due to rare or common species, using a novel model system, subterranean species. Subterranean species with extremely high endemism, challenge the view that common species constitute a backbone of species richness patterns. Although subterranean aquatic and terrestrial species differ in degrees of endemism and spatial patterns, our analyses consistently imply that also in such systems common species backbone the patterns.

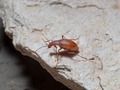

## 1 INTRODUCTION

Species richness, quantified as number of species per geographic unit, is not distributed evenly around the globe (Gaston & Blackburn, 2000; Zagmajster, Malard, Eme, & Culver, 2018). Studies of factors that shape species richness patterns (SRPs) explored either various environmental variables (for reviews see Beck et al., 2012; Field et al., 2009; Gaston & Blackburn, 2000; Stein, Gerstner, & Kreft, 2014) or species traits, in particular sizes of species' distribution ranges (Heegaard, Gjerde, & Saetersdal, 2013; Lennon, Beale, Reid, Kent, & Pakeman, 2011; Lennon, Koleff, Greenwood, & Gaston, 2004; Reddin, Bothwell, & Lennon, 2015; van Proosdij, Raes, Wieringa, & Sosef, 2016). In most taxonomic groups, species with small ranges (hereafter rare species) are more numerous than species with large ranges (hereafter common species) (Gaston, 1994a, 1996, 2003). The uneven frequencies of rare and common species invoke an intriguing question: Do SRPs emerge due to rare or common species?

This question has been studied in different taxa, mostly in vertebrates and plants (Gaston, 2008 and references therein), less so in invertebrates (Pearman & Weber, 2007; Reddin et al., 2015; Steck, Bürgi, Coch, & Duelli, 2007). Most of the studies suggested that common species shape the overall SRPs (Gaston, 2010; Jetz & Rahbek, 2002; Lennon et al., 2011, 2004). These observations led to appeals to include also common species into conservation strategies, as they “sustain” the SRPs (Gaston, 2010; Neeson et al., 2018).

These conservation implications should be considered with care. Species with the smallest (e.g., single‐site endemics) and the largest (e.g., everywhere present) ranges are not equally informative about SRPs. Indeed, after correction for the amount of information each species adds into the analyses, some studies yielded opposite conclusions that rare species are more important in shaping overall SRPs (Heegaard et al., 2013; Lennon et al., 2011, 2004; Reddin et al., 2015). The relative importance of rare and common species may change with the spatial scale of the analyses, but depend also on the studied taxon (Heegaard et al., 2013; van Proosdij et al., 2016). Overall, the contradictory results coupled with relative paucity of explicit studies, call for further testing of the hypothesis stating that common species shape SRPs.

Here, we approach this challenge using a novel and unique dataset comprised of species living exclusively in subterranean habitats. A striking characteristic of subterranean fauna is an exceptionally high proportion of extreme endemics, that is, species limited to few square kilometers or even one site (Eme et al., 2018; Niemiller & Zigler, 2013; Trontelj et al., 2009; Zagmajster et al., 2014). For example, in different parts of United States, 20%–45% of subterranean species are single‐site endemics (Christman, Culver, Madden, & White, 2005; Niemiller & Zigler, 2013), while in France, 38% of aquatic subterranean species have latitudinal linear range extents <3 km (Ferreira, Malard, Dole‐Olivier, & Gibert, 2007). In general, aquatic subterranean species with linear range extents above 200 km are an exception (Copilaș‐Ciocianu et al., 2017; Eme et al., 2018; Trontelj et al., 2009; Zagmajster et al., 2014). The extremely high proportion of rare species leads to an intuitive hypothesis that they are essential in formation of SRPs.

We used extensive distributional datasets of two groups, amphipods and beetles, living in subterranean habitats of the Western Balkans in southeastern Europe. The two taxonomic groups can be regarded as adequate models for aquatic and terrestrial faunas, as both represent a significant portion of species in the respective subterranean domains (Sket, Paragamian, & Trontelj, 2004). In addition, dispersal possibilities in aquatic or terrestrial habitats are different (Christman & Culver, 2001; Lamoreux, 2004; Porter, 2007). The SRPs have been well described for beetles, but not for amphipods (Bregović & Zagmajster, 2016; Zagmajster, Culver, Christman, & Sket, 2010; Zagmajster, Culver, & Sket, 2008). Hence, we first analyzed the overall SRPs of both groups and compared them to each other. Second, we investigated the contribution of rare and common species to overall SRPs for each taxon separately, using three analytical approaches. Third, we analyzed the shares of common and rare species in species‐rich cells within the taxonomic group. Finally, we discussed the conservation implications of the results.

## 2 METHODS

### 2.1 The study area and the dataset

The study area is located in the Western Balkans in southeastern Europe. It extends over the Dinarides (650 km in length, up to 150 km in width), and the Eastern parts of the Southern Calcareous Alps (60 km length and width; as in Bregović & Zagmajster, 2016). The geological substratum is mostly karstic (limestone and dolomite), with occasional interruptions of noncarbonate rocks. In this region, the number of animal species living exclusively in subterranean habitats is exceptionally high, leading to recognition of being a global hotspot in subterranean species richness (Culver et al., 2006; Zagmajster et al., 2014).

**Figure 1:**
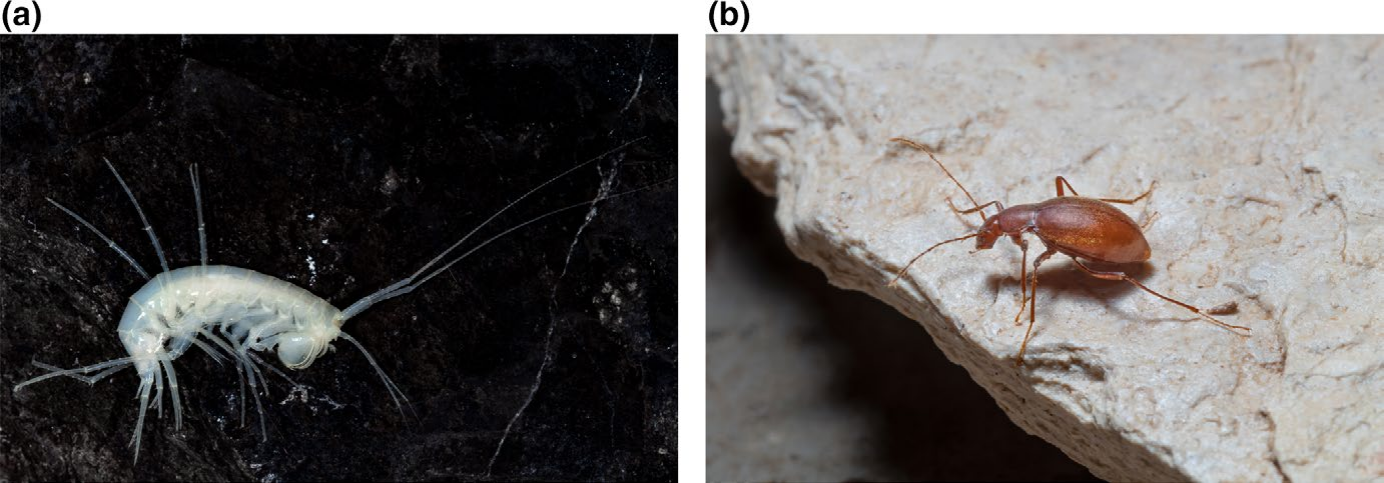
Representatives of the two taxonomic groups used in this study: Left is *Niphargus stenopus*—subterranean amphipod (photograph T. Delić) and right is *Spelaeodromus pluto*—subterranean beetle (photograph: P. Bregović)

Our study considered two taxonomic groups, subterranean amphipods belonging to family Niphargidae and subterranean beetles belonging to families Carabidae (Trechinae) and Leiodidae (Cholevinae; Figure 1). These groups present a substantial fraction of aquatic and terrestrial subterranean species in the region (Sket et al., 2004), so we consider them representative groups for the aquatic or terrestrial subterranean habitats (but see Christman et al., 2016).

**Figure 2:**
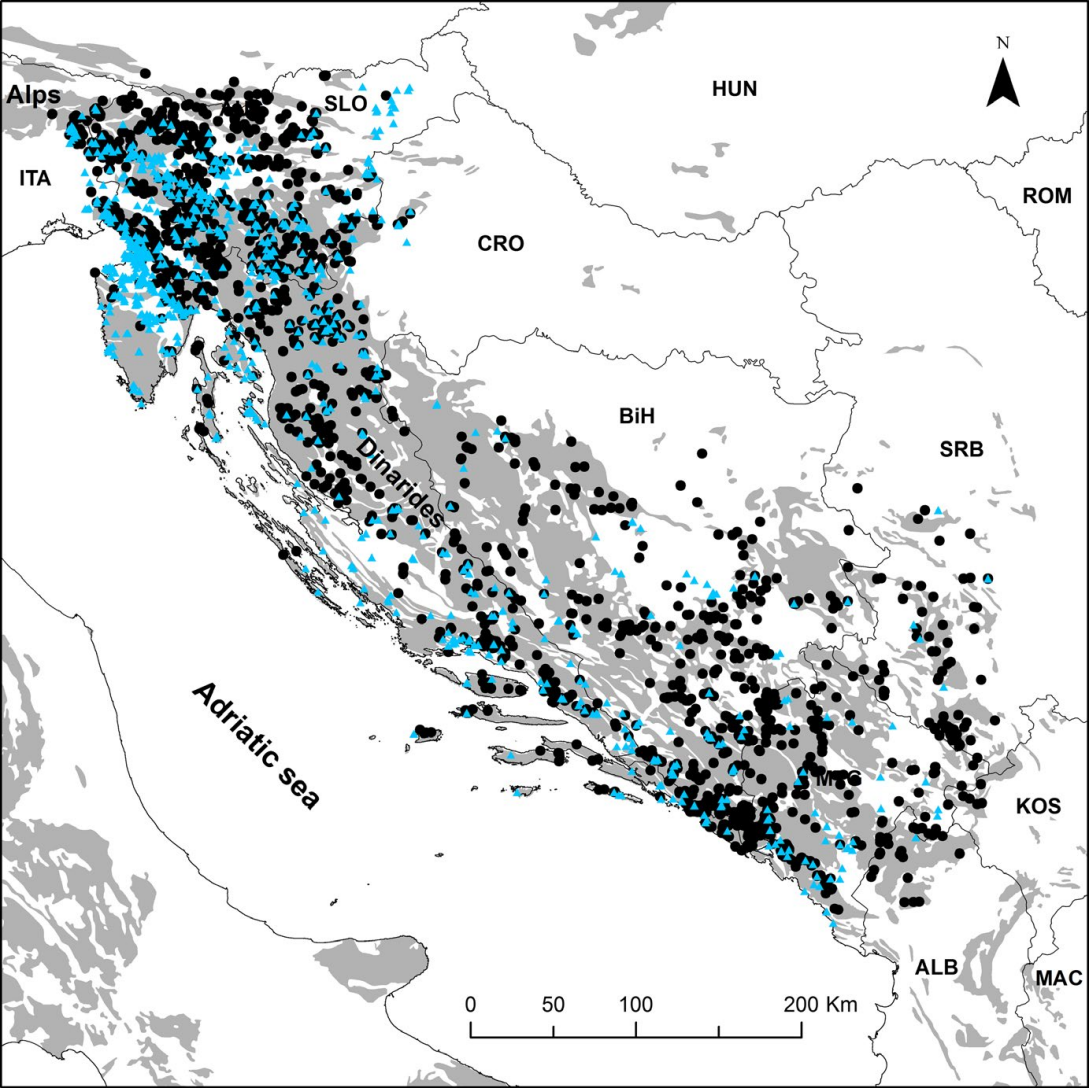
Distribution of localities (amphipods—blue triangles; beetles—black dots) used in analyses of subterranean species richness pattern in Western Balkans in southeastern Europe. Gray color denotes karstic areas (Lambert Conformal Conical Projection)

Distributional data consisted of point occurrence records, from published and our own unpublished distributional records organized in the relational database SubBioDB (Subterranean Biodiversity Database—http://subbio.net/db/) and European Groundwater Crustacean Dataset (Zagmajster et al., 2014). We considered only localities with at least 6 km positional accuracy (see Zagmajster et al., 2008 for details). The amphipod dataset consisted of 2,580 records (record = species + locality + reference), relating to 145 species and 1,760 localities, while the beetles dataset encompassed 8,236 records, relating to 431 species (156 Carabidae, 275 Leiodidae) and 2,523 localities (Figure 2).

### 2.2 Mapping species richness and range sizes

**Figure 3:**
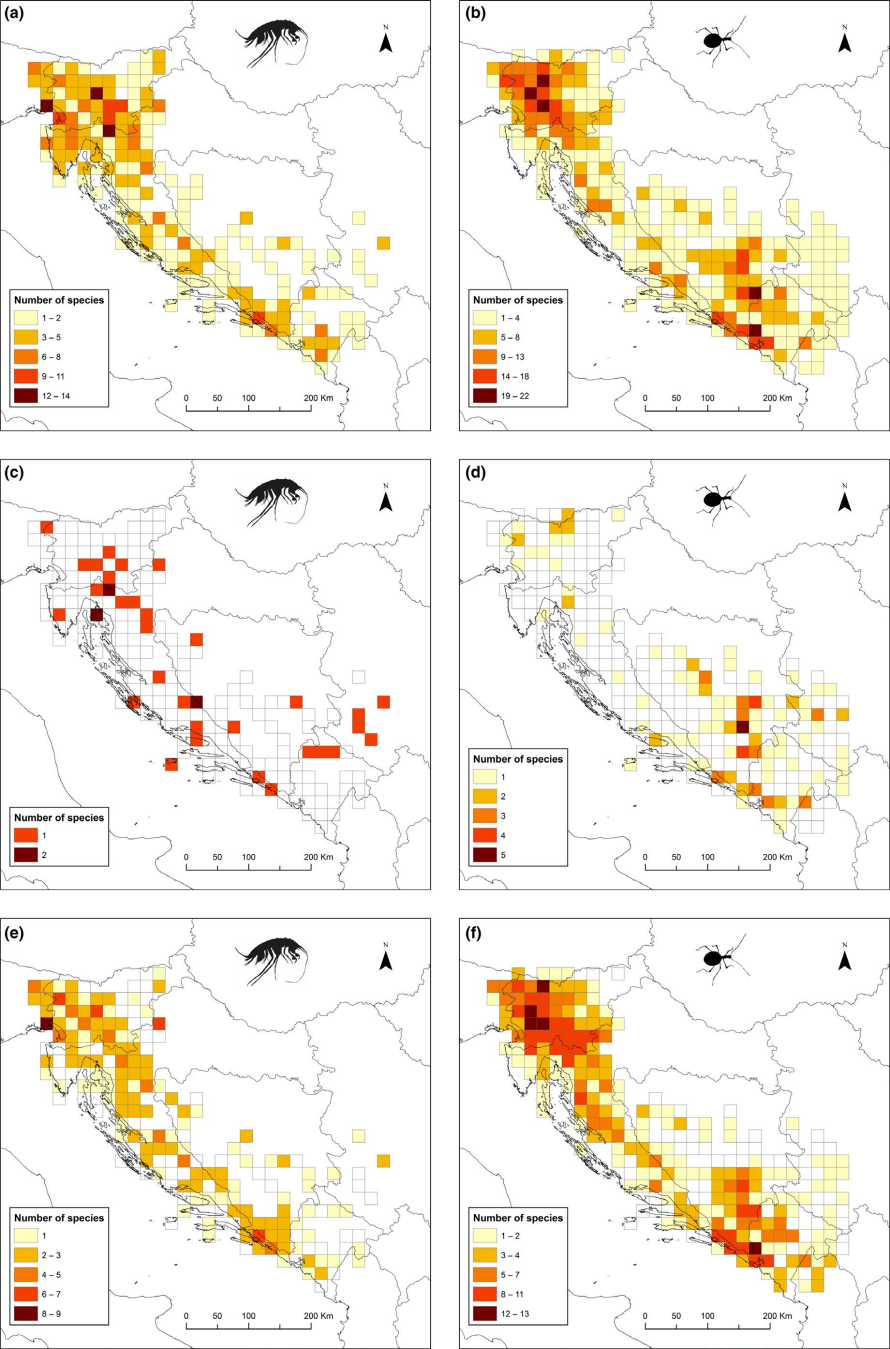
Species richness patterns of subterranean amphipods (left column) and beetles (right column) in Western Balkans, presented per 20 × 20 km grid cell size, for (a, b) all species, (c, d) rare species,and (e, f) common species. Class delimitation is defined according to the highest number of species per cell, as described in the text. Exception are rare species of amphipods, where categories are different due to small number of species per cell

The study area was overlain by a grid of 20 × 20 km cells, which has been shown to be the most appropriate for studies of subterranean SRPs in the region (Zagmajster et al., 2008), using the Lambert Conformal Conical Projection (central meridian 18°, parallels 42° and 46°; Figure 3). Species richness was quantified as number of species per cell. After exclusion of cells without records, the overall dataset comprised 191 and 262 grid cells for amphipods and beetles, respectively. We analyzed each taxon separately. To map SRPs, we categorized cells into five classes (following Bregović & Zagmajster, 2016; Zagmajster et al., 2008): the first class: >85% species of the richest cell, the second: 85%–60%, the third: 59%–40%, the fourth: 39%–20%, and the fifth: <20%. We defined cells of the first class as “hotspots” of species richness and the cells of the first and the second class as “species rich cells” (hereafter SRCs), having at least 60% of species numbers in the richest cell.

Range size of each species was calculated as maximum linear extent (MLE) and defined as linear distance between the two most distant point localities (Gaston, 1991, 1994b), and thereby, difficulties were avoided related to the use of two‐dimensional metrics (Gaston & Fuller, 2009). In determining MLE of the species, we considered all available species' occurrence records, including those occurring outside our study area (as in van Proosdij et al., 2016). The proportion of such species in our dataset was 13% in amphipods (19 species) and 0.9% in beetles (four species).

Rare and common species were determined using the quartile approach (Gaston, 1994a). Species within amphipods and beetles were separated into four quartiles, using MLE as a criterion. We regarded species of the first quartile as rare and the species of the fourth quartile as common (as in Lennon et al., 2004; Vazquez & Gaston, 2004).

Maps of SRPs and subsequent statistical analyses for each taxon were performed using three datasets: the overall dataset, the rare species dataset, and the common species dataset. All maps were prepared using ArcGIS 10.1 (ESRI, 2012), with Geospatial Modelling Environment used for grid data extraction (Beyer, 2014).

### 2.3 Statistical analyses

#### 2.3.1 Correlations among terrestrial and aquatic species richness patterns

We tested how well SRPs of amphipods and beetles correlate with each other. As values of species richness per cells contained many repetitive values in both groups, we calculated Kendall *τ*_b_ correlation coefficient, which performs well in such cases of many ties (Batt, Morley, Selden, Tingley, & Pinsky, 2017; Kendall, 1962; Sokal & Rohlf, 1995). In addition, this coefficient does not assume linear relationship among variables, making it suitable for our data.

#### 2.3.2 Reconstructing overall species richness patterns by stepwise species additions

For each taxon, we prepared two series of subsets according to species' range sizes. In every new subset, we added species stepwise, one‐by‐one, with respect to their range sizes. In the first series of subsets, species were ranked and added in range ascending order (the rarest species first). In the second series of subsets, species were ranked and added in descending order (the commonest species first). Then we assessed which order of species additions better reconstructs the overall SRPs, using correlation analysis (Lennon et al., 2011, 2004).

In order to investigate the contribution of rare and common species to overall SRPs for each taxon separately, we used three analytical approaches. Firstly, we calculated Kendall *τ*_b_ correlation coefficients between species richness of each subset and species richness of the overall dataset. The coefficients were plotted against the size of the subset (subset size as percentage of all species), allowing us to estimate whether range ascending or descending order more accurately represents the overall SRPs, as inferred from the values of Kendall *τ*_b_. As both datasets contained many single‐site species, which are equal in their range size (MLE = 0 km), these species were added into the ascending and descending subsets randomly. We performed 10,000 randomizations and then calculated the correlations as median value of correlations estimated from 10,000 repetitions (Reddin et al., 2015).

The significance was assessed using empirically determined *p*‐value, which was estimated from a null model. Null model was calculated from 10,000 randomly generated series of species subsets (Lennon et al., 2011).

In the second approach, the different amount of information brought by rare or common species subsets in the correlation analysis was considered, because results coming from the first approach may not have a biological meaning, but are a result of geometric or statistical causes of analyses and datasets (Lennon et al., 2011, 2004; Šizling, Šizlingová, Storch, Reif, & Gaston, 2009). To account for the problem of subsets' different informativeness, a weighting of subsets has been suggested using the expected binomial variance of subset richness pattern. In this approach, cumulative information content (CI) of subset was calculated as $\sum_{i=1}^{n}p_{i}\left(1-p_{i}\right)$, where *p_i_* is the proportion of sites occupied by *i*th species in the subset and *n* is the number of species in the subset (Lennon et al., 2011, 2004). With this approach, we can inspect how importance of rare and common species change with increasing volume of information of subsets, rather than percentage of species in every subset. In turn, we calculated the Kendall *τ*_b_ correlation coefficients as described above, but plotted them against CI of subsets rather than the size of the dataset as before. The significance was estimated using a null model, as described above.

In the third approach, we tested whether rare or common species predict better the overall SRPs (Vazquez & Gaston, 2004). For each taxon, we ran separate analyses on four subsets based on quartiles of MLE. The predictors were numbers of the selected species per cells, where species were drawn from the first and the fourth quartile according to their range size. The response variables in all four analyses were total numbers of species per cell. We used generalized linear models (GLM) that differ in the family of distribution, including Poisson distribution, or negative binomial distribution to account for overdispersion (Zuur, Hilbe, & Ieno, 2013; Zuur, Ieno, Walker, Saveliev, & Smith, 2009). Regression analysis was made using negative binomial GLM for rare species datasets and Poisson GLM for the common species datasets. The amount of variance explained in models was calculated using the pseudo *R* ^2^ (Dobson, 2002; Zuur et al., 2009), and model fit was assessed using Akaike information criterion corrected for small sample size (AICc; Burnham & Anderson, 2002).

To check for the spatial autocorrelation in these models, we calculated Moran's I coefficient on models' residuals (Dormann et al., 2007). Its size was calculated at arbitrary set 14 distance classes from the focal cell (as in Bregović & Zagmajster, 2016), and significance was tested using 10,000 permutations. Class sizes were defined to maximize the similarity in number of connections (Diniz‐Filho, Bini, & Hawkins, 2003). As spatial autocorrelation was present in residuals of all nonspatial GLM models, we ran the second set of models, which included also spatial components. We used spatial eigenvector mapping to produce spatial filters (i.e., eigenvectors; Diniz‐Filho & Bini, 2005). A truncation distance of 59 km (allowing the inclusion of two circles of neighboring cells around the focal cell) was used to create spatial filters in all models (as in Bregović & Zagmajster, 2016). Spatial filters were selected based on the following criteria: they minimized the spatial autocorrelation in model residuals (there were as many spatial filters as needed to completely remove autocorrelation from model residuals), maximized the regression multiple correlation coefficient, and had a significant correlation between response variable and each selected spatial filter (Diniz‐Filho & Bini, 2005; Griffith, 2003). Spatial filters were included as variables in the GLM models.

All statistical analyses were performed using R software, ver. 3.4.3 (R Core Team, 2017). Kendall *τ*_b_ correlation coefficients were calculated using the package “stats” and cor.test function (R Core Team, 2017), multimodel inference and model averaging were conducted with the package “MuMIn” (Barton, 2014), the fit of negative binomial GLMs calculated with the package “MASS” (Venables & Ripley, 2002). Moran's I and spatial filters were calculated using the SAM 4.0 software (Rangel, Diniz‐Filho, & Bini, 2010).

## 3 RESULTS

### 3.1 Comparison of species richness patterns between subterranean amphipods and beetles

The SRPs of subterranean amphipods and subterranean beetles differed. The correlation between the overall SRPs of both groups was low though significant (Kendall *τ*_b_ correlation = .17; *p* < .001).

The highest number of amphipod and beetle species per cell was 14 and 22, respectively. We identified three amphipod hotspots, counting at least 12 species (85% of the richest cell), all distributed in the northwest of the region. Hotspots for beetles, counting at least 19 species, were five, three of which were distributed in the northwest, while two were in the southeast (Figure 3). No hotspot cell was shared among amphipods and beetles.

The relationships between the taxa were roughly similar for the SRCs (having at least 60% species of the highest number). The number of SRCs in amphipods (eight cells, minimally nine species) was much lower than in beetles (19 cells, minimally 14 species; Figure 3). Seven amphipod SRCs were situated in northwest, and only one such cell was in the southeast. By contrast, ten beetle SRCs occurred in northwest and nine SRCs in southeast (Figure 3). Amphipods and beetles shared only one SRC, situated in the southeast of Dinarides (Figure 3).

### 3.2 Frequency distribution of range sizes

**Figure 4:**
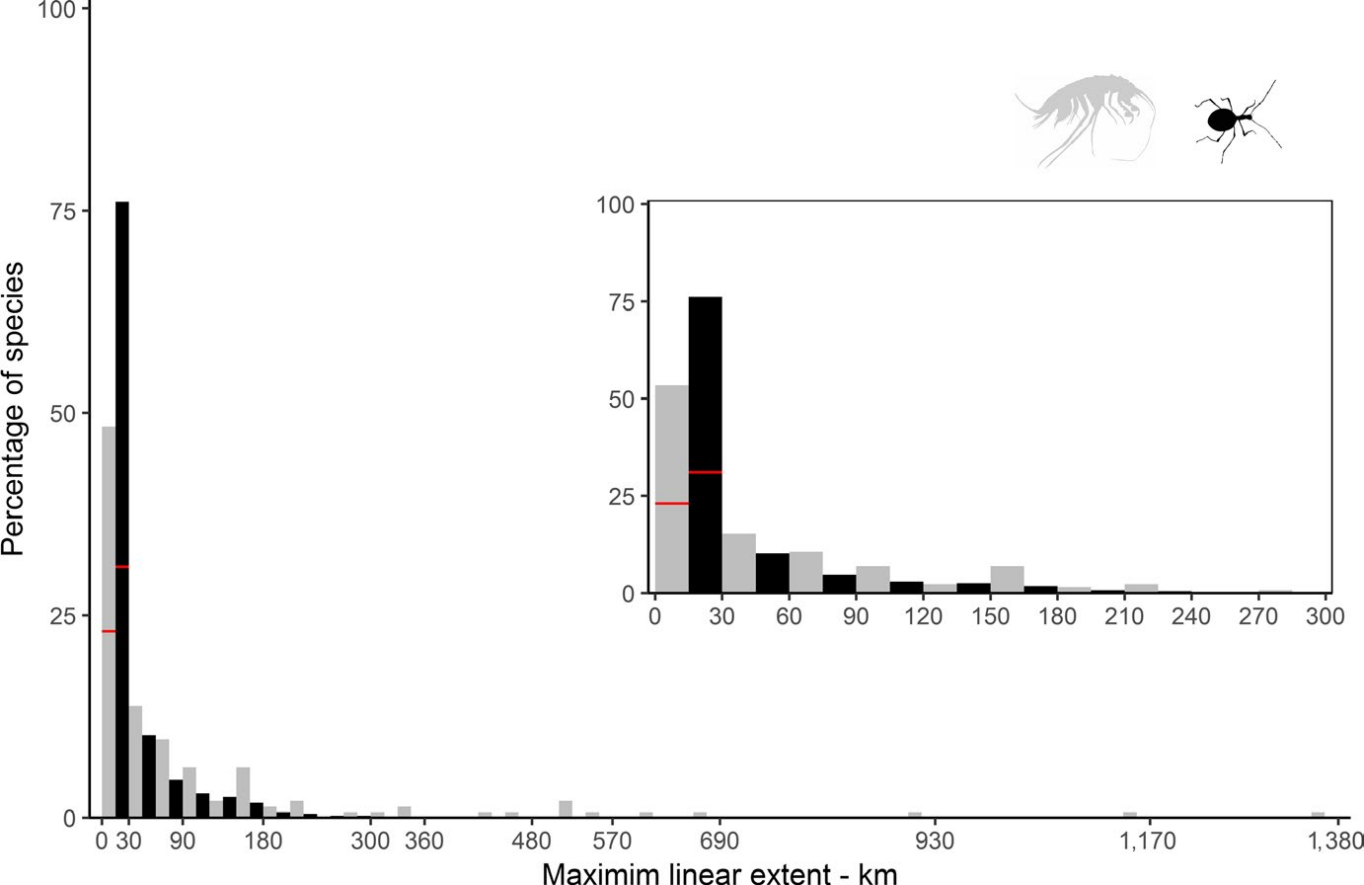
Range size frequency distribution for amphipods (gray) and beetles (black). Rectangle inside the plot is enlarged part of the histogram until 300 km. Red line indicates the percentage of single‐site species (23% in amphipods, 31% in beetles)

Frequency distribution of species' range sizes in both groups was strongly right skewed toward small ranges, with skewness coefficient 3.69 in amphipods and 2.64 in beetles (Figure 4). Ranges of amphipod species were on average larger than ranges of beetles (Table 1). The first quartile was dominated by single‐site species. MLE of the first quartile in amphipods spanned in a range 0–1 km, with 33 single‐site endemics species (23% of all the species). The number of single‐site endemics species in beetles was higher, counting 133 species (31% of all the species), with lower quartile value of 0 km. Species from the fourth quartile spanned their MLE between 109 and 1,376 km in amphipods and between 28 and 275 km in beetles.

**Table 1:** Number of species of subterranean amphipods and beetles in Western Balkans, in classes separated in quartiles of maximum linear extent (MLE)

Quartile	Amphipods		Beetles	
	Number (proportion) of species (%)	Maximum value of MLE (km)	Number (proportion) of species (%)	Maximum value of MLE (km)
1st quartile	37 (26)	1	133 (31)	0
2nd quartile	36 (25)	32	83 (19)	7
3rd quartile	36 (25)	109	107 (25)	28
4th quartile	36 (25)	1,376	108 (25)	275

Note

1st quartile is defined as rare species dataset, while the 4th quartile as common species dataset.

### 3.3 Reconstructing the overall species richness patterns

A visual inspection of SRPs of rare and common species (Figure 3c–f) shows that common species recovered the overall pattern better than rare species, both in amphipods and beetles.

**Figure 5:**
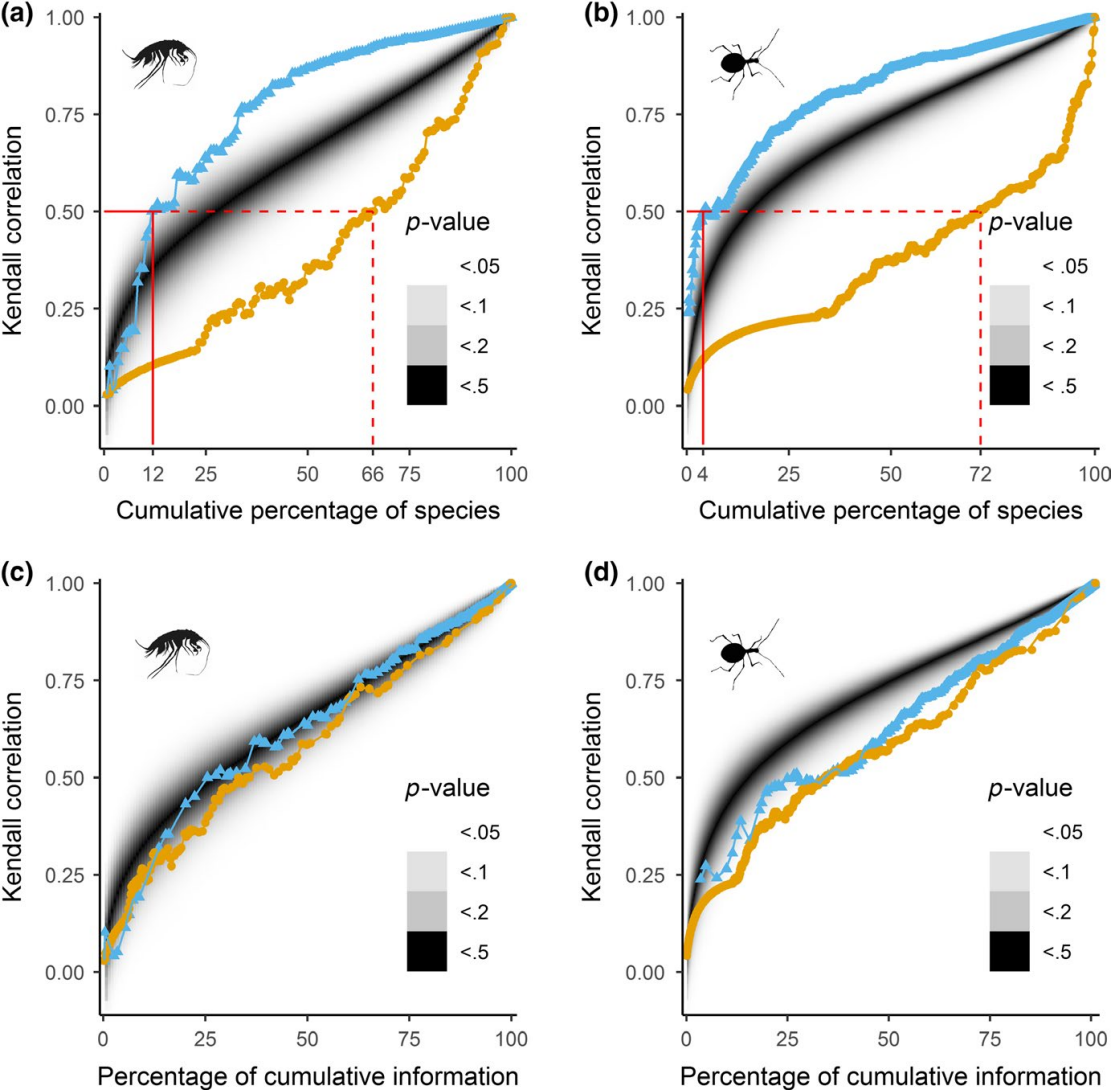
The correlation coefficients Kendall *τ*_b_ calculated between the species richness of subset and overall dataset, plotted against the percentage of species included in particular subset (a, b), or against the percentage of cumulative information in each subset, that is, expected binomial variance of subset's richness patterns (c, d). The subsets were built by addition of species one‐by‐one, ascendingly (yellow) or descendingly (blue) with respect to their MLEs. Single‐site species (MLE = 0 km), were included in the subsets at random; the plots show only median values of Kendall *τ*_b_ of 10,000 repetitions. The shaded region represents the null model generated by 10,000 random one‐by‐one additions of species regardless their MLEs. Red line indicates the percentage of species at which correlation coefficient exceeded .5 (solid line—descending order, dashed line—ascending order). The empirical *p*‐value indicates the probability of each point occurring at a distance from the median of the null distribution. (a, c) amphipods, (b, d) beetles

This is in agreement with the analyses that explored which order of stepwise species additions better represents the overall SRPs. The curve showing correlations between the SRPs of the subsets and the overall SRPs suggested that addition of species in descending order recovers the overall patterns faster and with fewer species (Figure 5, blue curve) than the addition of species in ascending order (Figure 5, yellow curve). Moreover, the descending order of species additions outperformed the random species addition in the null model (*p* < .05, Figure 5, gray). The correlation coefficients of species richness between subsets and the overall dataset exceeded .5 after adding 12% and 4% of the species in range descending order in amphipods and beetles, respectively (Figure 5a,b). This value was reached only when 66% (amphipods) and 72% (beetles) of species, sorted in range ascending order, were added (Figure 5). Despite these differences, the general conclusion that common species contribute more to SRPs was the same in amphipods and beetles.

In the second approach, after the correction for the information content brought by each subset, the results were ambiguous (Figure 5c,d). In both taxa, the curve of correlation coefficients increased faster when the species were added in range descending order (the commonest species first) than ascending order (the rarest species first). In amphipods, however, neither of curves deviated from the null model (*p* > .05, Figure 5c). This suggested that contribution of rare and common species to overall SRPs was indistinguishable in amphipods. Contrary to that, in beetles, both order of species additions reconstructed SRPs slower than random species addition in the null model, but the differences between the two curves were significant (*p* < .05, Figure 5d). Even though the curve for descending order reached higher correlation than ascending order at all the volume of information content, the two curves intersected at about .5 correlation (Figure 5d).

**Figure 6:**
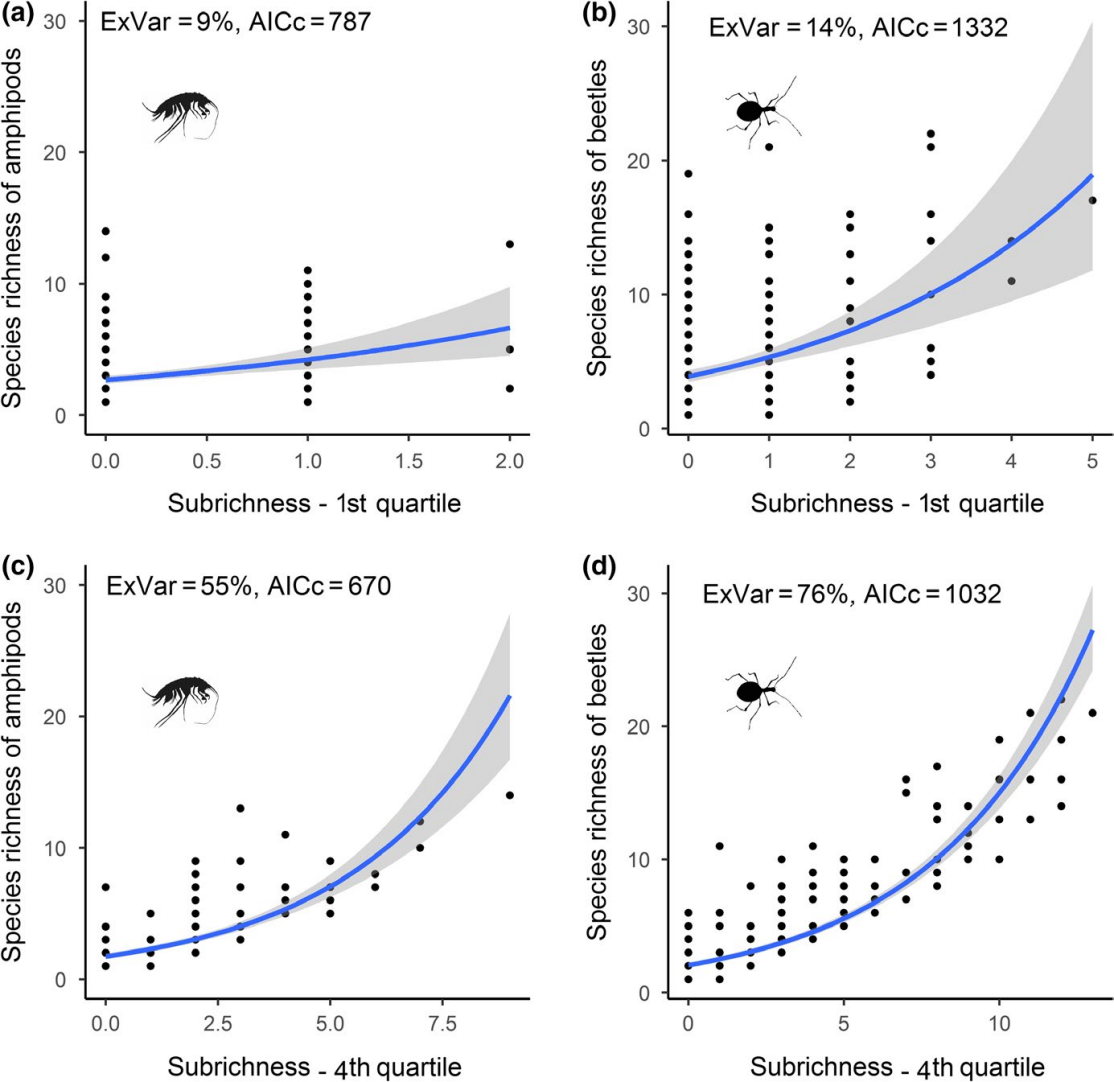
Plots of univariate regression between overall species richness and the number of species in 1st quartile—rare species (a, b) or 4th quartile—common species (c, d) in cells of the studied area. The blue line represents regression line according to generalized linear model; gray area is 95% confidence interval. AICc is Akaike's information criterion corrected for small sample size. (a, c) amphipods, (b, d) beetles

The results of regression analyses were consistent with results from stepwise species addition without corrected for information content. Common species predicted better the overall SRPs: They explained more variation of the overall SRPs and had better model fit (Table 2) and narrower confidence interval (Figure 6) than rare species. Spatial autocorrelation was present in all models' residuals (Figure S1). After its removal, the amount of explained variation of species richness increased in all spatial models, while the parameter estimates for the common and the rare species remained almost unchanged (Tables S1 and S2).

**Table 2:** Results of generalized linear models between species richness pattern using overall, common and rare species dataset in nonspatial and spatial models

Predictor/model	Amphipods		Beetles	
	Pseudo *R* ^2^	AICc	Pseudo *R* ^2^	AICc
Rare species/nonspatial model	0.09***	787	0.14***	1,332
Common species/nonspatial model	0.55***	670	0.76***	1,032
Rare species/spatial model	0.39***	733	0.58***	1,180
Common species/spatial model	0.71***	628	0.79***	1,010

Abbreviation: AICc, Akaike information criterion corrected for small sample size.

*** *p* < .001.

### 3.4 Proportion of rare and common species in SRCs

**Figure 7:**
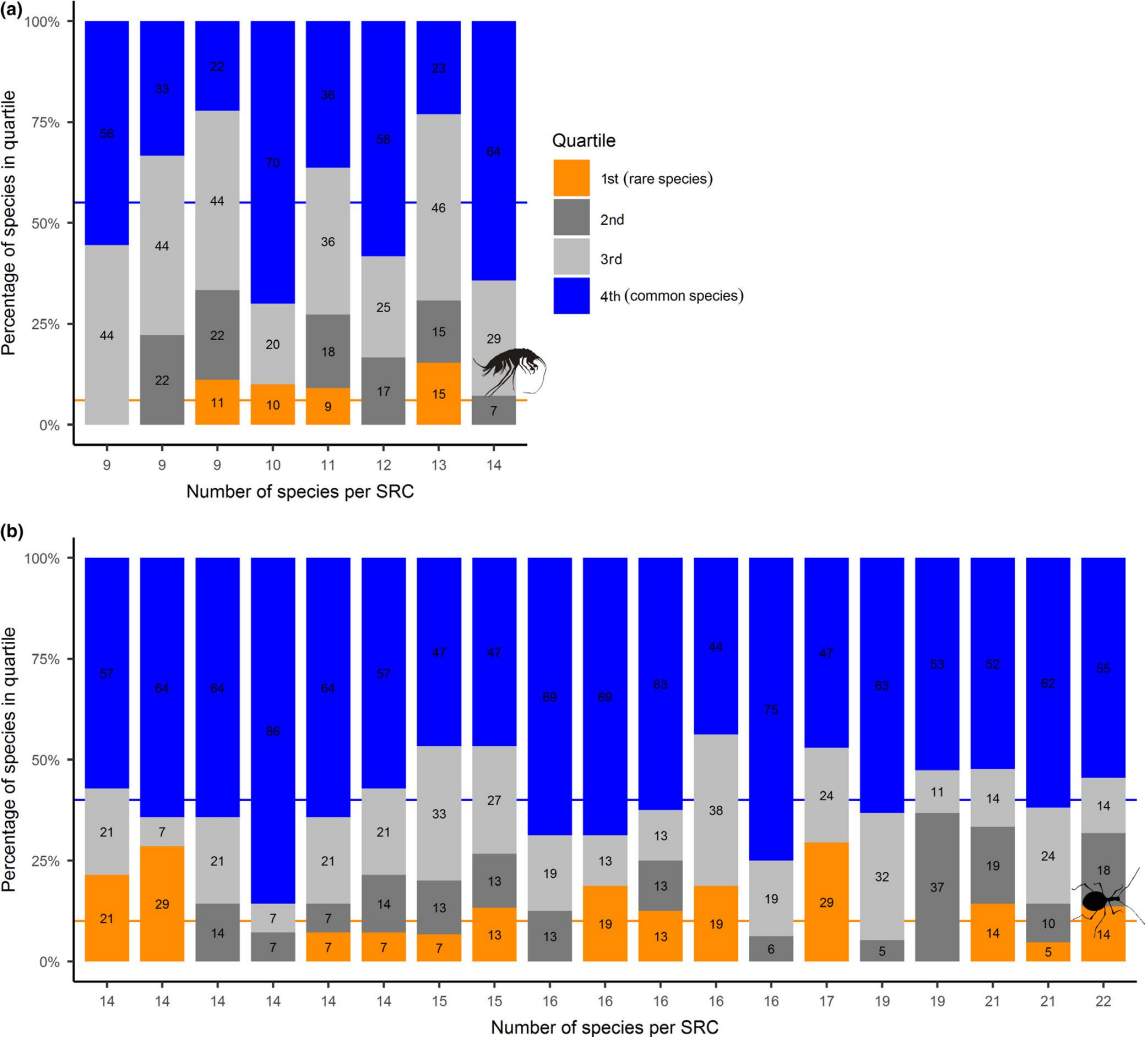
Contribution of species of different range quartiles to species‐rich cells (SRCs) for amphipods (a) and beetles (b). The *x*‐axis shows total number of species per cell, while *y*‐axis shows a percentage of species of certain range quartile within each SRC. Colors denote: Orange—1st quartile (rare species), dark gray—2nd quartile, light gray—3rd quartile, and blue—4th quartile (common species). The numbers refer to exact percentage of species within each SRC. Note that on average (blue line) common species represent higher share of species richness in SRCs than rare species (orange line). Note also that this effect is more pronounced in beetles

Overall, common species dominated in species composition of SRCs in comparison with rare species, although the relative share of either species differed between amphipods and beetles. However, in amphipods SRCs, rare and common species on average comprised 6% (0%–15%) and 45% (22%–64%) of all species, respectively. In contrast, in beetles SRCs rare and common species on average contributed 10% (0%–29%) and 60% (44%–86%) of species, respectively (Figure 7).

## 4 DISCUSSION

### 4.1 Reconstructing the overall species richness patterns

Species with small ranges dominated our datasets, with single‐site endemics counting up to one‐third of all species (23% in amphipods and 31% in beetles). However, different approaches of our analyses consistently indicated that common species constitute the backbone of the subterranean species richness patterns (SRPs) in the region. Our results therefore support the findings of other studies that common species contribute more to SRPs (Gaston, 2011; Jetz & Rahbek, 2002; Lennon et al., 2004). Considering the high proportion single‐site species had in both datasets and generally small ranges of subterranean species (Niemiller & Zigler, 2013; Zagmajster et al., 2010), our results were somewhat surprising and present a strong case for the greater contribution of common species to overall SRPs.

Considering the specific nature of subterranean species, which are generally narrow endemic, our results remain somewhat counterintuitive. What could be the reasons that despite high share of single‐site endemics, rare species contribute less to the overall SRPs than common species? One explanation is that rare species are distributed evenly across the study region (like in amphipods) or that their higher numbers aggregate in different cells than species richness hotspots (like in beetles). If rare species were predominantly found in species‐rich cells, then the correlation between rare species richness patterns and overall SRPs would be higher (Heegaard et al., 2013). Indeed, Zagmajster et al. (2010) have shown that some hotspot cells are sampled insufficiently, and perhaps more rare species could be found there. Two premises, however, suggest that more accurate datasets would not modify the conclusions. First, additional sampling could increase also the number of common species. Secondly, additional sampling could recategorize rare species into common ones, but not vice versa.

Noteworthy, it is important to keep in mind that the range size is a continuous variable (Gaston, 1994a). The cutoff between rare species and common species is not straightforward and always a bit arbitrary. Moreover, it should be taken into consideration that “commonness” is a relative term, inherent to each taxon and cannot be compared across the groups. In our study, the quartile approach sets the upper quartile value of amphipods as 109 km and beetles as only 24 km. Even though we defined this species as common in beetles, we are still dealing with fauna with extremely small ranges.

Our study has two important implications. First, common species may be considered as appropriate surrogates of overall species richness patterns also in subterranean habitats. Given that common species are more easily sampled than rare species (Gaston, 2011), and accurately reconstruct the overall SRPs, they could be used as surrogates for studies of overall SRPs (Gaston, 2008; Gaston & Fuller, 2008; Pearman & Weber, 2007). This surrogate purpose might have even higher taxonomic unit above species level (Mazaris, Kallimanis, Tzanopoulos, Sgardelis, & Pantis, 2010). Second, the results of our study need to be incorporated into studies that aim to understand the drivers behind SRPs, for species groups of different range sizes. Considering the revealed differences in relative contribution of the rare and common species to SRPs, the next step is to study the drivers behind those patterns separately in both groups of different range sizes (similarly as done in Jetz & Rahbek, 2002; Tetetla‐Rangel, Dupuy, Hernández‐Stefanoni, & Hoekstra, 2017).

### 4.2 Patterns of species richness and range size in the Western Balkans

We for the first time analyzed SRPs of the aquatic subterranean taxon along the Western Balkans. This sheds a new light on SRPs of the region, which were previously studied in the same detail only in terrestrial subterranean beetles (Bregović & Zagmajster, 2016; Zagmajster et al., 2008). Early study, based on country species lists, suggested that terrestrial and aquatic species richness peak in the southeast and northwest, respectively (Sket et al., 2004). More recent studies, based on spatially explicit species records challenged this view and identified two centers of terrestrial subterranean biodiversity (Bregović & Zagmajster, 2016; Zagmajster et al., 2008). We identified a convincing northwestern center of aquatic species richness, which are in line with previous studies (Sket et al., 2004), but found also some species‐rich cells in the southeast of the region.

Centers of terrestrial and aquatic subterranean biodiversity overlap only weakly. The two groups shared no hotspot cells (minimum 85% of maximum species richness), and only one species‐rich cell (SRC, minimum 60% of maximum species richness) in the southeast of the Dinarides. These results are in stark contrast to the findings of Niemiller and Zigler (2013) and their study on subterranean species richness in southeastern USA, using the grid cells of the same size. They found a strong correlation between patterns of aquatic and terrestrial subterranean species richness and identified the hotspots shared between them. Similarity in patterns of terrestrial and aquatic species richness could be expected due to shared geological events that influenced evolutionary histories of different taxa in our study region. However, the incongruent patterns suggest differences in dispersal abilities of the studied taxa and consequent differences in vicariance‐dispersal events (Culver, Pipan, & Schneider, 2009).

Our results suggest that subterranean amphipods are better dispersers than beetles. The biggest range of aquatic amphipod was five times greater than the biggest range of terrestrial beetle. This is in agreement with previous authors, who suggested higher dispersal possibilities in subterranean aquatic than in terrestrial species (Holsinger, 2000; Lamoreux, 2004). It is unclear whether the difference in dispersal possibilities derives from the differences in animal locomotion and active dispersal, or merely reflect differential connectivity of terrestrial and aquatic habitats (Christman & Culver, 2001; Porter, 2007). Generally speaking, the correlation between dispersal and range sizes is controlled by the magnitude of barriers between suitable habitat (Lester, Ruttenberg, Gaines, & Kinlan, 2007). The connectivity within subterranean realm is poorly understood. In theory, terrestrial species might disperse through better connected shallow subterranean habitats (Culver & Pipan, 2009), whereas aquatic species might disperse along surface rivers (Zakšek, Sket, Gottstein, Franjević, & Trontelj, 2009) or deep, unexplored phreatic connections (Konec, Delić, & Trontelj, 2016; Palandačić, Matschiner, Zupančič, & Snoj, 2012). In addition, previous studies suggested that connectivity of karst habitats was an important factor for subterranean SRPs (Bregović & Zagmajster, 2016). Likewise, the range size can be influenced by numerous biological factors such as interspecific competition (Beck et al., 2012; Kunin & Gaston, 1997; Slatyer, Hirst, & Sexton, 2013), which are poorly understood in subterranean habitats.

### 4.3 Conservation implications

Our study ran into the recently identified challenge in the conservation biology: Conserving rare species does not conserve overall species diversity (Bonn, Rodrigues, & Gaston, 2002), given that the areas of the highest species richness and the areas of the highest number of rare species do not always overlap (Orme et al., 2005). The problem is new to conservation of subterranean diversity, as hitherto studied hotspots contained also high shares of rare species (Christman et al., 2005; Niemiller & Zigler, 2013). All the conservation efforts could have focused on the species richest sites and rare species simultaneously (Niemiller, Taylor, & Bichuette, 2018).

The arguments for protection of rare species differ from arguments for protection of species‐rich cells and common species. Rare species have retained their appeal in conservation biology. These species have higher conservation value and are more likely to be threatened or already extinct (Pimm et al., 2014). Protection of areas with highest numbers of such species can be easily justified to decision makers. The arguments for consideration of common species in conservation planning are practical (Gaston & Fuller, 2008; Neeson et al., 2018). These species contribute substantially to ecosystem structure, ecosystem function, and macroecological patterns (Gaston, 2008, 2010, 2011; Winfree, Fox, Williams, Reilly, & Cariveau, 2015). The depletion of common species may be a serious, yet neglected problem, demonstrated by several recent studies (Inger et al., 2015; Petrovan & Schmidt, 2016).

The solution of this dilemma calls for the additional research. First, preserving ecosystem functions (Harvey, Gounand, Ward, & Altermatt, 2017) requires additional understanding what role in ecosystems play rare and common species, as well understanding interactions between different taxa (e.g., beetles from families Leiodidae vs. Carabidae) and ecological groups (e.g., aquatic vs. terrestrial). Second, an optimal conservation strategy should incorporate all available information into an analytic framework that will, in addition to species richness consider alternative metrics of biodiversity, such as rarity, weighted endemism, β diversity, and their relation to threat (Crain & Tremblay, 2014; Myers, Mittermeier, Mittermeier, da Fonseca, & Kent, 2000; Yu et al., 2017; Zhao, Li, Liu, & Qin, 2016). This analytic framework based on multiple criteria would possible consider common species, and not only rare species, in conservation planning of subterranean habitats.

## CONFLICT OF INTEREST

None declared.

## AUTHOR CONTRIBUTIONS

All authors contributed significantly to the conception of this study, P.B. conducted the analyses in line with discussion with C.F. and M.Z. All three authors contributed to data preparation and writing of the manuscript.

## Data Availability

Distribution maps for each species can be viewed via online interface on http://subbio.net/db/.
