# Supplementary material for: Contribution of rare and common species to subterranean species richness patterns
Source: Ecol Evol. 2019 Sep 30;9(20):11606–18. doi: 10.1002/ece3.5604 (PMC6822026; doi:10.1002/ece3.5604)
Supplement: Supplementary file 1 [file ECE3-9-11606-s001.docx]

**SUPPORTING INFORMATION**


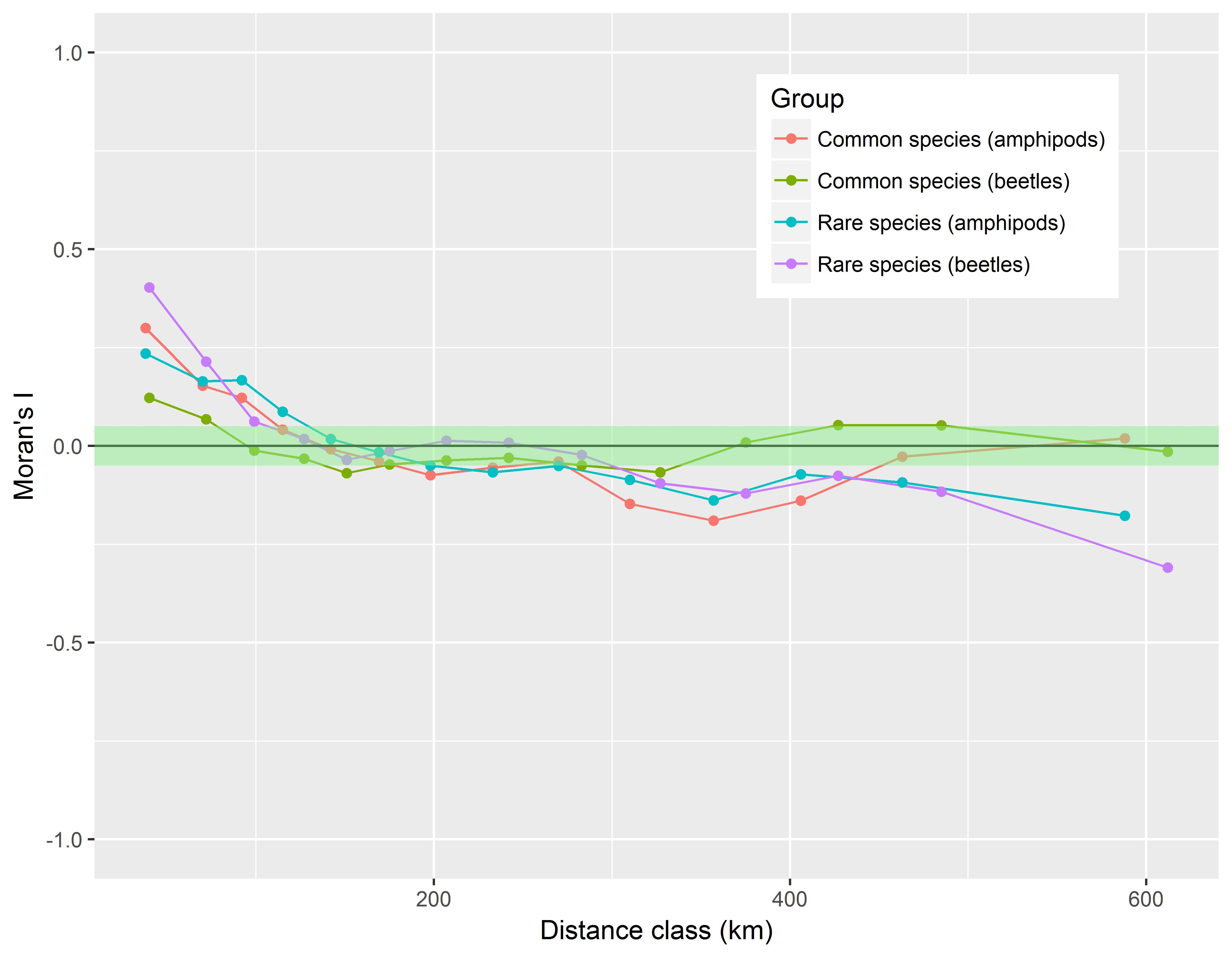


**Figure S1**. Moran’s I correlogram for residuals of non-spatial GLM models for rare and common species of amphipods and beetles, respectively. Green area represent the 0.05 threshold where SA is not significant.

**Table S1.** Model-averaged parameter estimates (E) and sums of Akaike weights (Σ*w_i_*) for variables explaining species richness in non-spatial and spatial models of amphipods and beetles. SR rare: species richness of rare species. SF1-SF9: spatial filters.

| Variables | Amphipods | | | | Beetles | | | |
| --- | --- | --- | --- | --- | --- | --- | --- | --- |
|  | Non-spatial models | | Spatial model | | Non-spatial model | | Spatial model | |
|  | E | Σ*w_i_* | E | Σ*w_i_* | E | Σ*w_i_* | E | Σ*w_i_* |
| Intercept | 0.989*** |  | 0.909*** |  | 1.359*** |  | 1.191*** |  |
| SR rare | 0.453*** | 1 | 0.460*** | 1 | 0.316*** | 1 | 0.356*** | 1 |
| SF1 |  |  | -3.517*** | 1 |  |  | 3.899*** | 1 |
| SF2 |  |  | 0.839 | 0.72 |  |  | -2.575*** | 1 |
| SF3 |  |  | 2.980*** | 1 |  |  | 3.909*** | 1 |
| SF4 |  |  | 2.129** | 0.98 |  |  | 3.427*** | 1 |
| SF5 |  |  | 1.261 | 0.84 |  |  | 2.761*** | 1 |
| SF6 |  |  | 1.267 | 0.85 |  |  | 2.046*** | 1 |
| SF7 |  |  |  |  |  |  | 2.367*** | 1 |
| SF8 |  |  |  |  |  |  | 1.040 | 0.83 |
| SF9 |  |  |  |  |  |  | 1.365 ˙ | 0.88 |

For E: ***p<0.001; **p<0.01; *p<0.05; ˙p<0.1

**Table S2.** Model-averaged parameter estimates (E) and sums of Akaike weights (Σ*w_i_*) for variables explaining species richness in non-spatial and spatial models of amphipods and beetles. SR common: species richness of common species. SF1-SF4: spatial filters.

| Variables | Amphipods | | | | Beetles | | | |
| --- | --- | --- | --- | --- | --- | --- | --- | --- |
|  | Non-spatial models | | Spatial model | | Non-spatial model | | Spatial model | |
|  | E | Σ*w_i_* | E | Σ*w_i_* | E | Σ*w_i_* | E | Σ*w_i_* |
| Intercept | 0.558*** |  | 0.519*** |  | 0.725*** |  | 0.720*** |  |
| SR common | 0.279*** | 1 | 0.273*** | 1 | 0.198*** | 1 | 0.194*** | 1 |
| SF1 |  |  | -2.783*** | 1 |  |  | 1.886*** | 1 |
| SF2 |  |  | -2.020** | 0.99 |  |  | -0.993 ˙ | 0.91 |
| SF3 |  |  | 1.639 ˙ | 0.91 |  |  | -1.248* | 0.92 |
| SF4 |  |  | -1.610* | 0.94 |  |  |  |  |

For E: ***p<0.001; **p<0.01; *p<0.05; ˙p<0.1
